# Supplementary material for: Low Hemoglobin Levels Are Associated with Reduced Psychomotor and Language Abilities in Young Ugandan Children
Source: Nutrients. 2022 Mar 30;14(7):1452. doi: 10.3390/nu14071452 (PMC9002834; doi:10.3390/nu14071452)
Supplement: Supplementary file 1 [file nutrients-14-01452-s001.zip › nutrients-1640902-supplementary.pdf]

Table of Contents

Table S1: Observational studies investigating associations between anemia and iron status and neurobehavioural outcomes in African pre-school children. 2

Table S2: Measures of motor and cognitive function used at age 15 months.. 5

Table S3. Principal component analysis, loadings on the 3 developmental components at 5 years of age 6

Table S4: Measures of motor and cognitive function used at age 5 years.. 6

Table S5. Maternal characteristics at enrolment. 7

Table S6: Distribution of individual developmental measures at 15 months and 5 years of age. 7

Table S7: Univariable and multivariable linear regression results for the associations between participant characteristics and developmental scores at 15 months.. 9

Table S8: Univariable and multivariable linear regression results for associations between participant characteristics and developmental scores at 5 years. 1

Table S9. Univariable and multivariable linear regression results for associations between maternal hemoglobin levels and anemia during pregnancy and developmental scores at 5 years. 3

Table S10: Univariable and multivariable linear regression results for the association between measures of iron status and anemia at two years of age and cognitive and motor scores at five years of age........ 4

Figure S1: Multivariable analyses between annual hemoglobin levels and developmental scores at 5 years.. 5

Table S1: Observational studies investigating associations between anemia and iron status and neurobehavioural outcomes in African pre-school children.

Author, year (country)	Sample	Study design	Age	Domain	Assessment tool	Definition of iron status	Results
Youssef, 2020 (Egypt)	226 children	Cross-sectional	2-6 years	IQ<br>Language	SBIS<br>Arabic language test	Not included	No association with IQ or language development.
Rothman, 2018 (South Africa)	750 infants	Cross-sectional	6 months	Psychomotor	KDI<br>Parent rating scale	Anemia: Hb<110 g/L ID: plasma ferritin<12 μg/L<br>IDA:ID+ Hb < 110 g/L	No association with psychomotor development.
Prado, 2017 (Ghana, Malawi, and Burkina Faso)	Mother-child pairs: Ghana (n=1023) and Malawi (n=675)<br>Child cohorts: Malawi (n=1385) and Burkina Faso (n=1122)	Cohort	18 months	Motor<br>Language	CDI<br>DMC<br>KDI	Anemia: Hb<110 g/L	No associations between maternal Hb or iron status during pregnancy and motor or language development at 18 months<br><br>Child Hb and iron status at six to nine months were associated with motor and language development at 18 months
Mireku, 2015 and 2016* (Benin)	636 mother-child pairs	Cohort	Maternal iron status and Hb at median 23 (19–26) weeks + cord blood ferritin and Hb at birth and neuroassessment in children at 1 year	Cognitive<br>Motor<br>Language	Mullen Scales of Early Learning	ID: SF<12 μg/L or SF 12-70 μg/L with inflammation: CRP >5 mg/L<br>Maternal anemia: Hb < 10 g/L<br>Child anemia birth: Hb <140 g/L<br>IDA: ID + anaemia	An inverted U-shaped association between maternal Hb and gross motor development with Hb 90–110 g/L being optimal for gross motor development.<br>No association between maternal iron status during pregnancy or cord blood ferritin and Hb levels and cognitive or language development at one year.
Gashu, 2016 (Ethiopia)	541 pre-school children	Cross-sectional	54-60 months	Cognitive	WPPSI-III, the school readiness test.	ID: SF<12μg/l<br>Anemia: Hb<110 g/l adjusted for altitude<br>IDA: ID + Hb<110 g/l	Anemia was associated with lower mean cognitive scores compared to normal Hb status<br><br>No association between ID/ IDA and cognitive development.

Angulo-Barroso, 2011 (Ghana, USA, China)	209 infants (49 from Ghana, 113 from USA and 47 from China)	Multisite cross-sectional	9-10 months	Motor	Pictorial milestone chart	IDA: Hb < 110 g/L + MCV <74 fl and/or RDW >14%<br>ID: Hb ≥ 110 g/L + MCV < 74 fl and/or RDW > 14%<br>IS: Hb ≥ 110 g/L, MCV ≥ 74 fl, and RDW ≤ 14%	IDA and ID were associated with lower motor function compared to sufficient iron status across the three study populations.
Olney, 2007 (Zanzibar)	771 children	Cross-sectional	5-19 months	Motor	A 20 mutually exclusive item code	IDA: Hb <100 g/L and ZnPP ≥90 mmol/mol heme<br>ID: ZnPP ≥90 mmol/mol heme<br>Anaemia: Hb <100 g/L	IDA and ID were associated with lower motor function compared to sufficient iron status.
Kariger, 2005 (Zanzibar)	646 children	Cross-sectional	6-18 months	Motor (walking and crawling)	Pictorial milestone chart	ID: ZnPP≥ 90 µmol/mol heme<br>IDA: Hb<100 g/L, ZnPP ≥90 mol/mol heme<br>IS: Hb≥ 100 g/L, ZnPP <90 µmol/mol heme<br>Anemia: Hb<100 g/L	Anemia and/or ID was associated with lower odds of walking compared to sufficient iron status<br>No association with crawling.
Bouhouch, 2016 (Morocco)	n=455 lead-exposed children (110 received iron, 116 received iron+ EDTA, 112 received EDTA, and 117 received placebo for 28 weeks)	RCT	3 to 14 years	Cognitive	(KABC-II, HVLT)	ID: SF <12 mg/L for children <5 years, SF <15 mg/L for children ≥5 years, or TfR >8.3 mg/L with (CRP ≤5 mg/L, α1-acid glycoprotein ≤51 g/L)<br>Anemia: Hb<11.0 g/dL for children <5 years, Hb<11.5 g/dL for children 5–11 years, Hb <12.0 g/dL for	No difference in cognitive scores between children who received iron supplementation or placebo.
Baumgartner, 2012 (South Africa)	n=288 (70 received iron+ placebo, 72 placebo + DHA/EPA, 73 iron+ DHA/EPA, and 73 placebo +	RCT	6 to 11 years	Cognitive	(HVLT, KABC)	ID: SF<15 µg/L excluding children with CRP >5 mg/L or ZnPP >70 µmol/mol or TfR >8.3 mg/L<br>Anemia: Hb <11.5 g/dL<br>IDA: anemia + SF<15 µg/L	Anemic children who received iron supplementation+ placebo had higher cognitive scores compared to children who received placebo + placebo<br><br>No difference in cognitive scores in children who received iron+ DHA/EPA compared to children who received placebo + placebo

	placebo for 8.5 months)						
Stoltzfus, 2001 (Zanzibar)	n=359<br>(183 received iron and 176 placebo for 12 months)	RCT	6 to 59 months	Language<br>Motor	Parents reported motor and language milestones	ID: SF<12 mg/L<br>Anemia: Hb<11g/dL<br>Severe anemia: Hb<7g/dL	Children who received iron had higher language scores compared to children who received placebo and children with baseline Hb<9g/dL who received iron had higher motor scores compared to children who received placebo.
Boivin, 1993 (Zaire)	N=47 (17 children received anthelminthics and iron, 7 only iron, 8 only anthelminthics, and 15 did not receive either intervention for 1 month)	RCT	Mean age for boys=7.7, SD=0.8 years) and for girls =8.0, SD =1.8 years	Cognitive	KABC	Anemia: Hb<12 g/dL	No difference in cognitive scores between children who received only iron supplementation or placebo.

CDI, communicative development inventory; DMC, Developmental milestones checklist; Hb, hemoglobin; ID, iron deficiency; IDA, iron deficiency anemia; IQ, intelligence quotient; IS, iron sufficiency; KDI, Kilifi Developmental Inventory; MCV, mean corpuscular volume; RDW, red blood cell distribution width; SBIS, Stanford Binet intelligence scale version four; WPPSI-III, Wechsler Preschool and Primary Scale of Intelligence; ZnPP, zinc protoporphyrin. * A single mother-child study with two publications separately evaluating the effects of maternal hemoglobin levels and iron status during pregnancy on developmental scores at one year.

Table S2: Measures of motor and cognitive function used at age 15 months

Name of test	Domain	Description of measure	Absolute scores (min, max)
Fine motor	Motor	Control of small hand-movements was assessed using 25 items such as building a tower with blocks and scribbling with a pen.	0, 32
Gross Motor	Motor	Control of the limbs assessed using 35 items such as kicking a ball or climbing onto a platform	0, 35
Self-control	Delay inhibition	This was assessed using two trials in which a biscuit (trial 1) or wrapped gift (trial 2) was presented to the child who was instructed not to open it until the assessor had finished what he/she was doing. Waiting time (in seconds) was recorded to a maximum of 150 seconds. The average time for the two trials was calculated.	0, 150
The A-not-B task	Contrast inhibition/ working memory	A biscuit was placed in one of two wells as the child watched and both wells were then covered with opaque cups. The board was taken out of sight for 10 seconds during which the child was distracted with a song. The board was then brought back, and the child asked to point to the well with the biscuit. The child was given the biscuit if she or he successfully located it. The location of the biscuit was switched to the other well after two consecutive correct responses. Ten trials were administered, and the number of correct responses recorded.	0, 10
Language interview	Language	The mother/guardian was interviewed on whether the infant produced common pre-speech items such as vowels (e.g., aa, aa), babble (e.g., ma, ma) or gestures (e.g., waving for ''bye'), spoke definite words, or names of common household objects (up to 11 items).	0, 22
Recognition of self and others	Socio-cognition	The mother/guardian was interviewed on whether the infant responded to his/her name or distinguished his mother and other familiar people from strangers (12 items).	0, 24
Self-care		The mother/guardian was asked about their child's behaviours such as how much she/he participated during dressing and feeding (15 items).	0, 30

Table S3. Principal component analysis, loadings on the 3 developmental components at 5 years of age

Measure	Component 1 (verbal and non-verbal IQ)	Component 2 (executive function)	Component 3 (motor ability)
**Block design**	0.65	-0.12	0.08
**Picture vocabulary scale**	0.57	0.17	-0.07
**Verbal fluency**	-0.15	0.59	0.13
**Picture search**	0.09	0.56	0.05
**Wisconsin card sort test**	0.32	0.41	-0.24
**Coin box**	-0.16	0.24	0.71
**Balancing on one leg**	0.31	-0.24	0.63

IQ, intelligence quotient. Component 1 (verbal and non-verbal IQ) was strongly positively correlated and with block design and picture vocabulary tests, component 2 (executive function) was positively correlated with verbal fluency, picture search and Wisconsin card sort tests and component 3 (motor ability) was strongly correlated with coinbox and balancing on one leg tests. The component scores are centred on zero with high scores indicating better and low scores worse development.

Table S4: Measures of motor and cognitive function used at age 5 years

PCA Components	Name of test	Domain	Description of measure	Absolute scores (min, max)
Verbal and non-verbal IQ	Block design	Non-verbal IQ	The measure is adapted from the British Ability Scales-third edition. The child is asked to copy and construct items with wooden blocks following a demonstration by the assessor.	0, 16
	Picture vocabulary scale	Verbal IQ	The measure is adapted from the Kilifi Vocabulary Test. The child is asked to point out and identify items from 24 black and white picture items familiar to them.	0, 24
Executive function	Verbal fluency	Working memory	The measure is adapted from the Developmental NEuroPSYchological Assessment. The child is asked to name items including foods and animals as fast as possible in a minute.	a
	Picture search	Selective attention	The measure is adapted from the Sky Search in Tests of Everyday Attention for Children. The child is presented with three A3 sheets each with a target picture on top and about 100 others at the bottom including copies of the target picture. The child is asked to locate as many copies of the target pictures as possible within 10 seconds.	b
	Wisconsin card sort test	Cognitive flexibility	The measure is adapted from Berg's card sort test. The child is given four playing cards of different suits and a pack of 12 cards and asked to sort the cards by number (block 1) and suit (block 2).	0,12

Motor function	Coin box	Fine motor function	The measure is adapted from the Kilifi Developmental Inventory. The child is asked to slot coins through a small opening on a coinbox within 20 seconds in two trials.	0, 20*
	Balancing on one leg	Gross motor function	The measure is adapted from the Movement Assessment Battery for Children. It entails timed attempts (two per leg) of balancing on one leg for one minute.	0, 60*

Abbreviations: PCA, principal components analysis; IQ, intelligence quotient; Min, minimum score; Max, maximum score. [a] One point is awarded for each correct name and a total score is calculated from the total correct names in a minute. [b] A total score is calculated from the number of target pictures identified within 10 seconds. *An average score is calculated after timed attempts of the tests. Block design and picture vocabulary tests loaded heavily on verbal and non-verbal IQ while verbal fluency, picture search, and Wisconsin card sort tests loaded heavily on executive function. Coin box and balancing on one leg tests loaded heavily on motor function. The resulting scores were centred on zero with higher scores representing better and lower scores worse development.

Table S5. Maternal characteristics at enrolment.

**Variable**	**Analysis for aim 1; maternal Hb during pregnancy, child Hb 12months and development at 15 months (*n*=933)**	**Analysis for aim 2; annual Hb and development at 5 years (*n*=726)**	**Analysis for aim 3; iron status at 2 years and development at 5 years (*n*=530)**
Maternal age (years), mean (SD)	24.2 (5.6)	24.2 (5.6)	24.5 (5.6)
Maternal education, n/total (%)			
Primary/none	494/930 (53.1)	393/724 (54.3)	295/528 (55.9)
Secondary	356/930 (38.3)	273/724 (37.7)	193/528 (36.6)
Tertiary	80/930 (8.6)	58/724 (8.0)	40/428 (7.6)
Maternal Parity, n/total (%)			
1	226/933 (24.2)	167/726 (23.0)	111/530 (20.9)
2-4	534/933 (57.2)	419/726 (57.7)	310/530 (58.5)
5+	173/933 (18.5)	140/726 (19.3)	109/530 (20.6)
Maternal treatment with albendazole, n/total (%)	475/933 (51.0)	366/726 (50.4)	272/530 (51.3)
Maternal treatment with praziquantel, n/total (%)	460/933 (49.3)	349/726 (48.1)	250/530 (47.2)

SD, standard deviation.

Table S6: Distribution of individual developmental measures at 15 months and 5 years of age.

**Measure**	***n***	**Obtained Median (interquartile range) scores**
At 15 months		

Fine motor	920	17 (16-18)
Gross motor	936	18 (16-19)
Psychomotor (fine motor + gross motor)	919	35 (32-37)
Language	933	15 (12-19)
Recognition of self and others	933	10 (10-10)
Self-care	933	18 (14-20)
Social cognition (recognition of self and others + self-care)	933	27 (24-30)
Self-control	805	1.5 (1-3.5)
A not B task	758	4 (2-6)
Executive function (A not B + self-control)	689	7 (5-9)
At 5 years		
Block design	804	9 (6-11)
Picture vocabulary scale	811	18 (16-20)
Verbal fluency	760	14 (8-20)
Picture search	811	4 (3-5)
Wisconsin card sort test	807	6 (3-10)
Coin box	807	10 (9-11)
Balancing on one leg	802	12 (7-20.3)

Table S7: Univariable and multivariable linear regression results for the associations between participant characteristics and developmental scores at 15 months.

Variables	*n*	Executive function β (95% CI)	*p* Value	*n*	Psychomotor function β (95% CI)	*p* Value	*n*	Social cognition β (95% CI)	*p* Value	n	Language β (95% CI)	*p* Value
Univariable linear regression results for the associations with developmental scores at 15 months												
Child characteristics												
Age at neuroassessment	687	0.01 (-0.13, 0.14)	0.94	916	0.14 (0.02, 0.26)	0.02	934	-0.02 (-0.21, 0.17)	0.84	934	0.09 (-0.03, 0.21)	0.14
Sex (female)	688	-0.04 (-0.19, 0.11)	0.59	918	-0.16 (-0.29, -0.03)	0.01	936	0.18 (-0.03, 0.38)	0.09	936	0.02 (-0.11, 0.15)	0.72
Malaria parasitemia at 1st annual visit (Positive)	686	0.25 (-0.11, 0.60)	0.17	915	-0.39 (-0.69, -0.09)	0.01	933	-0.39 (-0.87, 0.08)	0.10	933	-0.16 (-0.46, 0.13)	0.28
Clinical malaria episodes at 1 year of age	688	-0.04 (-0.16, 0.07)	0.47	918	-0.11 (-0.21, -0.02)	0.02	936	-0.04 (-0.13, 0.06)	0.43	936	0.004 (-0.09, 0.10)	0.94
Any worm infections at 1st annual visit	659	-0.04 (-0.52, 0.44)	0.88	889	0.10 (-0.30, 0.51)	0.62	905	-0.16 (-0.82, 0.50)	0.64	905	-0.32 (-0.74, 0.09)	0.13
Child treatment with albendazole (placebo)	687	-0.03 (-0.18, 0.11)	0.66	916	0.01 (-0.12, 0.14)	0.90	934	-0.11 (-0.31, 0.10)	0.30	934	-0.07 (-0.20, 0.06)	0.28
Nutritional status												
Stunting	680	0.10 (-0.12, 0.32)	0.39	907	-0.53 (-0.72, -0.35)	<0.001	925	-0.31 (-0.60, -0.01)	0.04	925	-0.28 (-0.47, -0.09)	0.003
Underweight	688	-0.17 (-0.43, 0.09)	0.21	918	-0.55 (-0.77, -0.32)	<0.001	936	-0.34 (-0.70, 0.02)	0.07	936	-0.23 (-0.45, -0.002)	0.05
Wasting	680	0.01 (-0.38, 0.37)	0.98	907	-0.55 (-0.87, -0.24)	0.001	925	-0.12 (-0.63, 0.39)	0.65	925	-0.09 (-0.41, 0.24)	0.60
Maternal characteristics at enrolment												
Maternal age (years)	689			919			933			933		
14-24		Reference			Reference			Reference			Reference	
25-34		-0.04 (-0.20, 0.12)			0.10 (-0.04, 0.24)			0.18 (-0.04, 0.39)			0.10 (-0.03, 0.24)	
35+		-0.16 (-0.47, 0.14)	0.31*		0.18 (-0.08, 0.45)	0.07*		0.16 (-0.27, 0.59)	0.13*		-0.06 (-0.32, 0.21)	0.49*
Maternal education	686			916			934			934		

**Variables**	***n***	**Executive function β (95% CI)**	***p* Value**	***n***	**Psychomotor function β (95% CI)**	***p* Value**	***n***	**Social cognition β (95% CI)**	***p* Value**	**n**	**Language β (95% CI)**	***p* Value**
Primary/none		0.16 (-0.11, 0.43)			-0.29 (-0.53, -0.06)			0.18 (-0.20, 0.56)			-0.15 (-0.39, 0.08)	
Secondary		0.19 (-0.09, 0.46)			-0.09 (-0.33, 0.16)			0.31 (-0.08, 0.70)			0.07 (-0.16, 0.32)	
Tertiary		Reference	0.50*		Reference	0.001*		Reference	0.99*		Reference	0.01*
Parity	689			919			933			933		
1		Reference			Reference			Reference			Reference	
2-4		-0.01 (-0.19, 0.17)			-0.02 (-0.18, 0.13)			0.09 (-0.15, 0.34)			-0.03 (-0.19, 0.12)	
5+		0.01 (-0.22, 0.23)	0.99*		-0.05 (-0.25, 0.15)	0.63*		0.14 (-0.17, 0.45)	0.37*		-0.03 (-0.23, 0.17)	0.77*
Albendazole treatment in pregnancy (placebo)	689	-0.05 (-0.20, 0.09)	0.48	919	-0.05 (-0.18, 0.08)	0.43	933	-0.10 (-0.30, 0.10)	0.34	933	0.02 (-0.10, 0.15)	0.71
Praziquantel treatment in pregnancy (placebo)	689	-0.15 (-0.29, -0.002)	0.05	919	0.03 (-0.10, 0.16)	0.62	933	-0.10 (-0.30, 0.11)	0.36	933	0.05 (-0.08, 0.18)	0.46
Household social economic status	675			902			919			919		
1 (lowest)		0.09 (-0.34, 0.53)			-0.05 (-0.43, 0.33)			-0.22 (-0.83, 0.40)			-0.37 (-0.76, 0.01)	
2		0.08 (-0.32, 0.49)			-0.31 (-0.67, 0.05)			-0.01 (-0.58, 0.56)			-0.20 (-0.56, 0.16)	
3		0.09 (-0.24, 0.42)			-0.31 (-0.60, -0.02)			-0.11 (-0.57, 0.36)			-0.27 (-0.57, 0.02)	
4		0.11 (-0.19, 0.50)			-0.05 (-0.34, 0.25)			0.26 (-0.21, 0.74)			-0.08 (-0.37, 0.22)	
5		0.16 (-0.19, 0.50)			-0.09 (-0.39, 0.21)			0.06 (-0.42, 0.55)			-0.07 (-0.38, 0.23)	
6 (highest)		Reference	0.87*		Reference	0.04*		Reference	0.13*		Reference	0.01*
Multivariable linear regression results for the associations with developmental scores at 15 months**												

Variables	*n*	Executive function β (95% CI)	*p* Value	*n*	Psychomotor function β (95% CI)	*p* Value	*n*	Social cognition β (95% CI)	*p* Value	n	Language β (95% CI)	*p* Value
Child characteristics												
Age at neuroassessment	624	0.04 (-0.10, 0.19)	0.57	835	0.16 (0.04, 0.28)	0.01	846	-0.01 (-0.22, 0.19)	0.91	850	0.10 (-0.03, 0.22)	0.14
Sex (female)	621	-0.07 (-0.23, 0.09)	0.41	831	-0.25 (-0.38, -0.12)	<0.001	846	0.15 (-0.07, 0.37)	0.19	846	-0.02 (-0.15, 0.12)	0.82
Malaria parasitemia at 1st annual visit (Positive)	621	0.17 (-0.22, 0.56)	0.39	831	-0.21 (-0.52, 0.11)	0.20	846	-0.37 (-0.88, 0.15)	0.16	846	-0.04 (-0.36, 0.28)	0.80
Clinical malaria episodes at 1 year of age	624	-0.05 (-0.17, 0.08)	0.47	835	-0.06 (-0.16, 0.03)	0.18	850	-0.02 (-0.12, 0.08)	0.75	850	0.05 (-0.05, 0.15)	0.33
Nutritional status												
Stunting	621	0.07 (-0.17, 0.31)	0.57	831	-0.51 (-0.70, -0.31)	<0.001	846	-0.32 (-0.63, 0.004)	0.05	846	-0.27 (-0.47, 0.08)	0.01
Underweight	629	-0.22 (-0.50, 0.07)	0.14	842	-0.48 (-0.72, -0.25)	<0.001	857	-0.31 (-0.69, 0.08)	0.12	857	-0.14 (-0.38, 0.10)	0.26
Wasting	621	0.003 (-0.41, 0.41)	0.99	831	-0.49 (-0.82, -0.16)	0.004	846	-0.04 (-0.59, 0.52)	0.89	846	-0.02 (-0.36, 0.33)	0.93
Maternal characteristics at enrolment												
Maternal age (years)	621			831			846			846		
14-24		Reference			Reference			Reference			Reference	
25-34		-0.01 (-0.18, 0.16)			0.06 (-0.07, 0.20)			0.16 (-0.08, 0.39)			0.08 (-0.07, 0.22)	
35+		-0.25 (-0.58, 0.08)	0.31*		0.24 (-0.04, 0.53)	0.09*		0.18 (-0.29, 0.65)	0.18*		0.01 (-0.29, 0.30)	0.49*
Maternal education	621			831			846			846		
Primary/none		0.15 (-0.13, 0.44)			-0.21 (-0.44, 0.03)			0.24 (-0.16, 0.64)			-0.07 (-0.32, 0.18)	
Secondary		0.18 (-0.11, 0.47)			-0.03 (-0.27, 0.21)			0.36 (-0.04, 0.76)			0.12 (-0.13, 0.37)	
Tertiary		Reference	0.57*		Reference	0.01*		Reference	0.76*		Reference	0.08*

Variables	*n*	Executive function β (95% CI)	*p* Value	*n*	Psychomotor function β (95% CI)	*p* Value	*n*	Social cognition β (95% CI)	*p* Value	n	Language β (95% CI)	*p* Value
Praziquantel treatment in pregnancy (placebo)	621	-0.06 (-0.22, -0.09)	0.45	831	0.04 (-0.09, 0.17)	0.58	846	-0.11 (-0.32, 0.11)	0.33	846	0.07 (-0.06, 0.21)	0.29
Household social economic status	621			831			846			846		
1 (lowest)		0.08 (-0.38, 0.54)			0.07 (-0.32, 0.45)			-0.35 (-1.01, 0.30)			-0.36 (-0.77, 0.04)	
2		0.13 (-0.31, 0.57)			-0.24 (-0.61, 0.13)			-0.14 (-0.76, 0.48)			-0.16 (-0.55, 0.23)	
3		0.08 (-0.28, 0.44)			-0.19 (-0.49, 0.11)			-0.15 (-0.66, 0.35)			-0.23 (-0.55, 0.08)	
4		0.07 (-0.28, 0.43)			0.01 (-0.29, 0.31)			0.19 (-0.32, 0.69)			-0.07 (-0.38, 0.25)	
5		0.14 (-0.23, 0.50)			-0.09 (-0.39, 0.22)			-0.11 (-0.62, 0.41)			-0.11 (-0.43, 0.21)	
6 (highest)		Reference	0.97*		Reference	0.35*		Reference	0.17*		Reference	0.03*

CI, confidence interval. Stunting, underweight and wasting were defined as height-for-age Z scores <-2 SD, weight-for-age Z scores <-2 SD and weight-for-height Z scores <-2 SD respectively. *P value for linear trend. **Multivariable analyses were only conducted for variables that were associated with developmental scores in univariable analyses. Multivariable models were adjusted for age, sex, hemoglobin levels, stunting, socioeconomic status, maternal education, helminthic infections and malaria parasitaemia.

Table S8: Univariable and multivariable linear regression results for associations between participant characteristics and developmental scores at 5 years.

**Variables**	***n***	**Verbal/nonverbal IQ β (95% CI)**	***p* Value**	**Executive function β (95% CI)**	***p* Value**	**Motor ability β (95% CI)**	***p* Value**
Univariable linear regression results for the associations with developmental scores at 5 years							
Child characteristics							
Age at iron measurement	530	-0.05 (-0.19, 0.09)	0.49	-0.08 (-0.21, 0.06)	0.25	-0.02 (-0.13, 0.09)	0.73
Sex (female)	724	0.34 (0.15, 0.54)	0.001	-0.05 (-0.24, 0.13)	0.57	0.18 (0.02, 0.33)	0.03
Malaria parasitaemia during 1-5 years of follow-up	726	-0.36 (-0.65, -0.08)	0.01	-0.33 (-0.59, -0.06)	0.01	-0.30 (-0.52, -0.07)	0.01
Clinical malaria episodes during 1-5 years of follow-up	722	-0,08 (-0.19, 0.03)	0.15	-0.05 (-0.16, 0.05)	0.31	-0.04 (-0.13, 0.05)	0.38
Any worm infections during 1-5 years of follow-up	726	-0.25 (-0.50, 0.004)	0.05	-0.32 (-0.56, -0.08)	0.01	-0.11 (-0.31, 0.09)	0.28
Child treatment with albendazole	724	-0.08 (-0.28, 0.12)	0.43	0.06 (-0.13, 0.24)	0.56	-0.08 (-0.24, 0.07)	0.29
Child nutritional status at 5 years							
Stunting	710	-0.38 (-0.61, -0.16)	0.001	-0.73 (-0.94, -0.52)	<0.001	-0.13 (-0.31, 0.05)	0.15
Underweight	714	-0.40 (-0.73, -0.07)	0.02	-0.61 (-0.93, -0.30)	<0.001	-0.29 (-0.55, -0.02)	0.04
Wasting	713	-0.06 (-0.44, 0.32)	0.75	-0.12 (-0.48, 0.24)	0.51	-0.11 (-0.42, 0.19)	0.47
Maternal characteristics at enrolment							
Maternal age (years)	726						
14-24		Reference		Reference		Reference	
25-34		0.19 (-0.02, 0.41)		0.12 (-0.08, 0.32)		-0.03 (-0.20, 0.14)	
35+		-0.13 (-0.52, 0.27)	0.50*	-0.07 (-0.45, 0.30)	0.67*	-0.12 (-0.43, 0.20)	0.46*
Maternal education	724						
Primary/none		-0.66 (-1.03, -0.29)		-0.80 (-1.14, -0.45)		0.05 (-0.25, 0.35)	
Secondary		-0.35 (-0.74, 0.03)		-0.40 (-0.75, -0.04)		0.10 (-0.20, 0.41)	
Tertiary		Reference	<0.001*	Reference	<0.001*	Reference	0.86*
Parity	726						
1		Reference		Reference		Reference	
2-4		0.09 (-0.16, 0.33)		-0.04 (-0.27, 0.19)		0.11 (-0.08, 0.31)	
5+		-0.22 (-0.52, 0.08)	0.19*	-0.22 (-0.51, 0.07)	0.15*	0.08 (-0.17, 0.32)	0.49*
Albendazole treatment in pregnancy (placebo)	726	0.01 (-0.19, 0.21)	0.94	0.01 (-0.17, 0.20)	0.88	0.10 (-0.06, 0.26)	0.21

Variables	*n*	Verbal/nonverbal IQ β (95% CI)	*p* Value	Executive function β (95% CI)	*p* Value	Motor ability β (95% CI)	*p* Value
Praziquantel treatment in pregnancy (placebo)	726	0.04 (-0.16, 0.24)	0.69	0.06 (-0.12, 0.25)	0.41	0.07 (-0.09, 0.23)	0.37
Household social economic status	713						
1 (lowest)		-0.37 (-0.90, 0.17)		-0.68 (-1.18, -0.17)		-0.05 (-0.49, 0.38)	
2		-0.01 (-0.54, 0.53)		-0.26 (-0.77, 0.25)		-0.08 (-0.51, 0.36)	
3		-0.16 (-0.57, 0.25)		-0.26 (-0.65, 0.13)		0.16 (-0.17, 0.49)	
4		-0.02 (-0.44, 0.39)		-0.06 (-0.45, 0.33)		0.20 (-0.13, 0.53)	
5		0.37 (-0.06, 0.80)		0.32 (-0.09, 0.72)		0.16 (-0.19, 0.51)	
6 (highest)		Reference	0.002*	Reference	<0.001*	Reference	0.43*
Multivariable linear regression results for the associations with developmental scores at 5 years**							
Child characteristics							
Sex (female)	471	0.48 (0.25, 0.71)	<0.001	0.02 (-0.20, 0.24)	0.83	0.29 (0.10, 0.48)	0.003
Malaria parasitemia during 1-5 years of follow-up	471	-0.46 (-0.80, -0.11)	0.01	-0.18 (-0.51, 0.15)	0.28	-0.20 (-0.49, 0.09)	0.17
Any worm infections during 1-5 years of follow-up	471	0.18 (-0.13, 0.48)	0.26	0.02 (-0.27, 0.31)	0.90	0.06 (-0.19, 0.31)	0.65
Nutritional status at 5 years							
Stunting	471	-0.07 (-0.33, 0.19)	0.62	-0.53 (-0.79, -0.28)	<0.001	-0.03 (-0.26, 0.19)	0.76
Underweight	474	-0.18 (-0.55, 0.19)	0.34	-0.35 (-0.71, 0.01)	0.06	-0.17 (-0.48, 0.13)	0.27
Maternal characteristics at enrolment							
Maternal education	471						
Primary/none		-0.49 (-0.95, -0.03)		-0.47 (-0.91, -0.03)		0.24 (-0.14, 0.62)	
Secondary		-0.02 (-0.49, 0.44)		-0.09 (-0.54, -0.35)		0.30 (-0.09, 0.68)	
Tertiary		Reference	<0.001*	Reference	0.001*	Reference	0.61*
Household social economic status	471						
1 (lowest)		-0.41 (-1.05, 0.22)		-0.37 (-0.98, 0.24)		0.08 (-0.45, 0.61)	
2		0.02 (-0.57, 0.62)		-0.03 (-0.60, 0.54)		0.08 (-0.42, 0.57)	
3		-0.24 (-0.71, 0.23)		-0.09 (-0.54, 0.36)		0.26 (-0.14, 0.65)	
4		-0.22 (-0.70, 0.25)		0.03 (-0.42, 0.49)		0.19 (-0.20, 0.59)	
5		0.09 (-0.39, 0.59)		0.29 (-0.17, 0.76)		0.16 (-0.25, 0.57)	
6 (highest)		Reference	0.09*	Reference	0.02*	Reference	0.72*

CI, confidence interval. Stunting, underweight and wasting were defined as height-for-age Z scores <-2 SD, weight-for-age Z scores <-2 SD and weight-for-height Z scores <-2 SD respectively. *P value for linear trend. ** Multivariable analyses only included variables that were associated with developmental scores in the univariable analyses. Multivariable models were adjusted for age, sex, any

moderate anemia event, iron deficiency, stunting, socioeconomic status, maternal education, helminthic infections and malaria parasitemia.

Table S9. Univariable and multivariable linear regression results for associations between maternal hemoglobin levels and anemia during pregnancy and developmental scores at 5 years.

**Developmental domain**	***n***	**Univariable model β (95% CI)**	***p* Value**	***n***	**Multivariable model* β (95% CI)**	***p* Value**
Maternal hemoglobin levels and developmental scores at 5 years						
Verbal and non-verbal IQ	724	0.05 (-0.02, 0.12)	0.14	693	0.04 (-0.03, 0.11)	0.29
Executive function	724	0.06 (-0.004, 0.13)	0.07	693	0.05 (-0.01, 0.12)	0.10
Motor ability	724	-0.02 (-0.08, 0.03)	0.42	693	-0.03 (-0.09, 0.02)	0.25
Mild maternal anemia and developmental scores at 5 years						
Verbal and non-verbal IQ	724	-0.09 (-0.30, 0.12)	0.39	693	-0.06 (-0.26, 0.15)	0.58
Executive function	724	-0.12 (-0.32, 0.07)	0.22	693	-0.12 (-0.31, 0.07)	0.21
Motor ability	724	0.08 (-0.09, 0.24)	0.37	693	0.09 (-0.08, 0.26)	0.29
Moderate maternal anemia and developmental scores at 5 years						
Verbal and non-verbal IQ	724	-0.15 (-0.42, 0.12)	0.28	693	-0.10 (-0.37, 0.18)	0.48
Executive function	724	-0.10 (-0.36, 0.16)	0.46	693	-0.06 (-0.32, 0.19)	0.63
Motor ability	724	0.05 (-0.17, 0.27)	0.64	693	0.10 (-0.12, 0.33)	0.37

CI, confidence interval. Mild maternal anemia was defined as hemoglobin levels <11 g/dL and moderate maternal anemia was defined as hemoglobin levels <10 g/dL. All haemoglobin measures were adjusted for high altitude (1000 m above sea level). *The multivariable models were adjusted for age at developmental assessment, sex, stunting, socioeconomic status, maternal education, helminthic infections, malaria parasitaemia and any moderate child anaemia event during follow up.

Table S10: Univariable and multivariable linear regression results for the association between measures of iron status and anemia at two years of age and cognitive and motor scores at five years of age

**Iron parameter**	***n***	**Univariable model, β (95% CI)**	***p* Value**	***n***	***BRINDA adjusted model β (95% CI)**	***p* Value**
**Verbal/non-verbal IQ**						
ID	490	0.11 (-0.14, 0.36)	0.37	413	0.22 (-0.05, 0.49)	0.11
IDA	472	-0.08 (-0.42, 0.26)	0.66	422	0.02 (-0.24, 0.29)	0.85
Ferritin (μg/L)	495	-0.09 (-0.21, 0.02)	0.43	413	-0.01 (-0.02, 0.002)	0.12
Transferrin (g/L)	517	0.17 (-0.03, 0.36)	0.09	431	0.12 (-0.09, 0.33)	0.27
sTfR (mg/L)	518	-0.12 (-0.29, 0.04)	0.15	429	-0.02 (-0.06, 0.01)	0.15
Hepcidin (μg/L)	516	-0.07 (-0.16, 0.03)	0.16	426	-0.01 (-0.01, 0.003)	0.22
**Executive Function**						
ID	490	0.29 (0.05, 0.53)	0.02	413	0.06 (-0.20, 0.31)	0.67
IDA	472	-0.08 (-0.41, 0.25)	0.65	422	-0.05 (-0.30, 0.20)	0.68
Ferritin (μg/L)	495	-0.13 (-0.24, -0.02)	0.02	413	-0.01 (-0.02, 0.003)	0.22
Transferrin (g/L)	517	0.22 (0.04, 0.41)	0.02	431	0.15 (-0.05, 0.36)	0.15
sTfR (mg/L)	518	-0.03 (-0.19, 0.13)	0.72	429	-0.01 (-0.04, 0.02)	0.43
Hepcidin (μg/L)	516	-0.12 (-0.21, -0.03)	0.01	426	-0.01 (-0.01, 0.003)	0.17
**Motor Ability**						
ID	490	0.13 (-0.07, 0.33)	0.21	413	0.06 (-0.16, 0.29)	0.57
IDA	472	0.08 (-0.19, 0.35)	0.58	422	0.07 (-0.14, 0.29)	0.50
Ferritin (μg/L)	495	-0.08 (-0.17, 0.01)	0.09	413	-0.01 (-0.02, -0.002)	0.02
Transferrin (g/L)	517	0.01 (-0.15, 0.16)	0.91	431	0.03 (-0.14, 0.21)	0.71
sTfR(mg/L)	518	-0.09 (-0.23, 0.04)	0.18	429	-0.01 (-0.04, 0.01)	0.35
Hepcidin (μg/L)	516	-0.03 (-0.11, 0.04)	0.38	426	-0.01 (-0.01, 0.001)	0.07

CI, confidence interval; sTfR, soluble transferrin receptor; ID, iron deficiency; IDA, iron deficiencyanemiaa. ID was defined as plasma ferritin < 12 μg/L in the absence of inflammation or < 30 μg/L in the presence of inflammation (CRP >5 mg/L); Iron deficiency anemia was defined as the presence of both iron deficiency and mild anemia (hemoglobin <11 g/dL). All hemoglobin measures were adjusted for change in altitude (1000 m above sea level). The multivariable models were adjusted for age at iron measurement, sex, stunting, inflammation, helminthic infections, socioeconomic status, maternal education, and malaria parasitemia. Ferritin, sTfR, hepcidin and CRP levels were natural log (ln) transformed to normalize their distribution. * Iron profiles are regression corrected for inflammation and malaria using the BRINDA approach [26].

Figure S1: Multivariable analyses between annual hemoglobin levels and developmental scores at 5 years

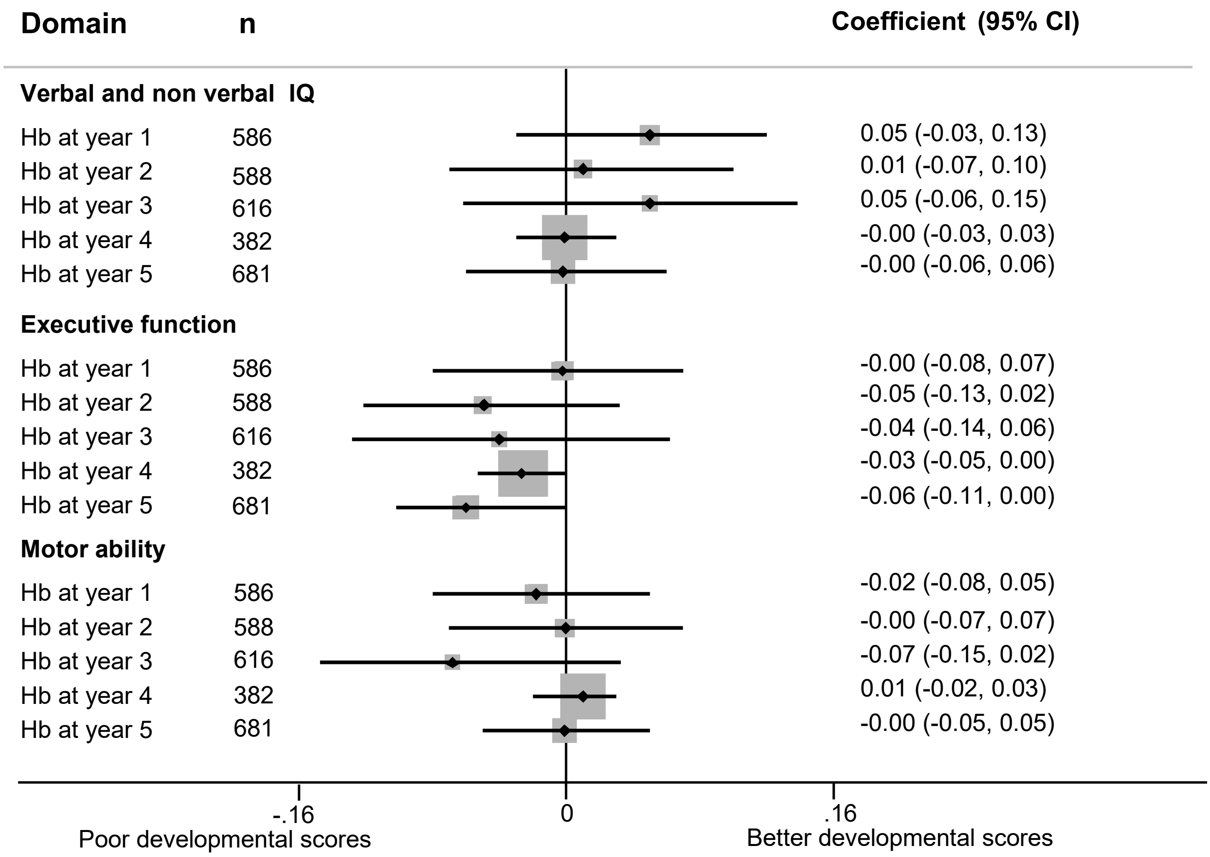

CI, confidence interval; Hb, hemoglobin levels. A forest plot of the multivariable regression analyses between annual hemoglobin levels and developmental scores at five years adjusted for age at developmental assessment, sex, stunting, socioeconomic status, maternal education, helminthic infections and malaria.
